# Effects of haloperidol, olanzapine, ziprasidone, and PHA‐543613 on spatial learning and memory in the Morris water maze test in naïve and MK‐801‐treated mice

**DOI:** 10.1002/brb3.764

**Published:** 2017-07-11

**Authors:** Houxu Ning, Dong Cao, Haidong Wang, Bing Kang, Shiping Xie, Yujing Meng

**Affiliations:** ^1^ Department of Psychiatry of Chinese Medicine Affiliated Nanjing Brain Hospital of Nanjing Medical University Nanjing China; ^2^ Department of Psychiatry Affiliated Nanjing Brain Hospital of Nanjing Medical University Nanjing China; ^3^ Department of Psychiatry Nanjing Medical University Nanjing China

**Keywords:** haloperidol, mice, MK‐801, Morris water maze, olanzapine, PHA‐543613, RRID: SCR_014289, ziprasidone

## Abstract

**Introduction:**

Cognitive impairment is the core symptom of schizophrenia, significantly impacting the functional outcome. Improvement of cognitive function has been an important aspect of the treatment of schizophrenia. Therefore, this study is to demonstrate the effects of first‐generation antipsychotic haloperidol, second‐generation antipsychotic olanzapine and ziprasidone, and alpha‐7 nicotinic acetylcholine receptor agonist PHA‐543613 on spatial learning and memory.

**Material and Methods:**

C57BL/6 mice received intraperitoneal injections of haloperidol (2 mg/kg), olanzapine (2.5 mg/kg), ziprasidone (2 mg/kg), and PHA‐543613 (1 mg/kg), and cognitive dysfunctions were induced by MK‐801 (0.1 mg/kg). Morris water maze was used for investigating the effects of all agents.

**Results:**

Mk‐801 significantly increased the mean escape latency to the platform and decreased the number of platform area crossings. Ziprasidone had no effect on the mean escape latency to platform and the number of platform area crossings in naïve mice, but haloperidol, olanzapine, and PHA‐543613 did not. Haloperidol and olanzapine significantly increased the mean escape latency to platform and decreased the number of platform area crossings, while ziprasidone and PHA‐543613 did not. All the agents had no effect on swimming speed.

**Conclusions:**

Ziprasidone and alpha‐7 nicotinic acetylcholine receptor agonist PHA‐543613 might be helpful in the treatment of CIAS.

## 1 INTRODUCTION

Schizophrenia is a severe mental disorder and affects about 1% of the worldwide population (Rössler, Joachim Salize, van Os, & Riecher‐Rössler, 2005). Cognitive impairment associated with schizophrenia (CIAS) is a core symptom domain besides positive symptoms and negative symptoms, which impairs quality of life and impacts roughly 70% of patients (Barch & Ceaser, 2012; Tyson, Laws, Flowers, Mortimer, & Schulz, 2008). Spatial learning and memory is one of the fundamental components of cognition, and schizophrenia patients perform impaired spatial and episodic memory (Barch, 2005; Nuechterlein et al., 2008). Current antipsychotic treatments, which are the mainstay therapeutic option for schizophrenia patients, demonstrate little benefits on CIAS (Young & Geyer, 2015). Nevertheless, treatment of CIAS might significantly sway the patient's functional outcome more than other symptoms.

The hypofunction of glutamatergic receptors is the pathophysiology of schizophrenia of the glutamatergic hypothesis, and the *N*‐methyl‐d‐aspartate receptor (NMDAR) antagonists, such as dizocilpine (MK‐801), phencyclidine, and ketamine, produce schizophrenia‐like behavior and cognitive deficits (Gaspar, Bustamante, Silva, & Aboitiz, 2009; Lobellova et al., 2013; Meltzer et al., 2013). Acute treatment with MK‐801 is extensively utilized to establish animal model of cognitive impairment, such as spatial learning and memory. While schizophrenia is a chronic psychiatric illness, chronic treatment with MK‐801 in rodents was shown to cause prolonged memory impairment (Karamihalev, Prickaerts, & van Goethem, 2014), which should be more consistent with CIAS (Zemanova et al., 2013).

Haloperidol is a typical antipsychotic with a high affinity for dopamine D_2_ receptors that are the targets of antipsychotic drugs, which was shown to impair the spatial learning memory in Morris water maze (MWM) in mice (Xu, Yang, & Rose, 2012). Olanzapine is an atypical antipsychotic agent widely used in treatment of schizophrenia and schizoaffective disorder (Volavka et al., 2002). Olanzapine, like other atypical antipsychotics (i.e., clozapine and risperidone), has less selective activity on various neuronal receptors, including antidopaminergic, antiserotonergic, and antimuscarinic (Bymaster et al., 1996). Ziprasidone, another atypical antipsychotic, exhibits a high affinity with serotonin 5HT_1A_, serotonin 5HT_2A_, and serotonin 5HT_2C_ receptors (Caley & Cooper, 2002) apart from dopamine D_2_ and D_3_ receptors. A previous study found that ziprasidone might improve performance in MWM in MK‐801‐treated mice, whereas there was no effect in naïve mice (Tanyeri et al., 2015). PHA‐543613 is a highly selective alpha‐7 nicotinic acetylcholine receptor agonist with cognitive enhancer potential, but produced little efficacy in terms of the memory deficit of MK‐801‐treated rats in the previous study (Bali et al., 2015).

In this present study, we attempt to investigate the influences of haloperidol, olanzapine, ziprasidone, and PHA‐543613 on spatial learning and memory both in naïve and subchronic MK‐801 mouse model of cognitive impairment using MWM test.

## 2 METHODS

### 2.1 Animals

A total of 100 male C57BL/6 mice (20–25 g) aged 7 weeks upon arrival to the laboratory was used as experimental subjects. All animals (5 per cage) were housed in laboratory under 12 hr light cycle from 7 a.m. to 7 p.m. at 21 ± 1.5°C with food pellets and tap water available ad libitum for 7 days before experimentation. All procedures were approved by Nanjing Medical University Ethics Committee for Experimental Animal Research.

### 2.2 Morris water maze test

The MWM consisted of a circular pool with a diameter of 960 mm and 50 cm height filled water (21 ± 1.5°C) to 40 cm deep and painted white. As previously described (Vorhees & Williams, 2006; Yu et al., 2015), the mice were required to find the location of a hidden platform below the surface of the water, and underwent four trials per day from different release positions that were varied systematically for six consecutive days. During the acquisition phase of the first 5 days, if the mouse failed to escape on the platform within 60 s, it was guided to climb on platform, and the video‐tracking system was used to record the latency of finding the platform. During the acquisition phase, the mean swimming speed was recorded. On the sixth day of probe test, the platform was removed and the mouse was allowed to search the maze for 60 s, whereas the number of platform area crossings was measured. The swimming path of the mice was analyzed using ANY‐maze software (ANY‐maze, Version 4.98, Stoelting Co, RRID: SCR_014289).

### 2.3 Drugs and treatment of mice

MK‐801 and PHA‐543613 were purchased from Sigma‐Aldrich (St. Louis, MO, USA). Haloperidol was purchased from Hunan Dongting Pharm. Co., Ltd (Hunan, China). Olanzapine was purchased from Lily del Caribe. Inc. Ziprasidone was purchased from Pfizer Pharmaceuticals Limited. All of the drugs were dissolved in saline and freshly prepared and administered in a volume of 0.1 ml per 10 g body weight. The control group received the same volume of vehicle. All the mice were administered saline or MK‐801 (0.1 mg/kg) intraperitoneally for 10 days before MWM test. Haloperidol (2 mg/kg, HAL), olanzapine (2.5 mg/kg, OLA), ziprasidone (2 mg/kg, ZIP), and PHA‐543613 (1 mg/kg, PHA) were administered to the naïve mice and MK‐801‐treated mice 30 min before the acquisition phase and probe test. The dosages and paradigms of injections were performed according to previous studies (Karamihalev et al., 2014; Mutlu, Ulak, Celikyurt, Akar, & Erden, 2011; Nilsson, Markinhuhta, & Carlsson, 2006; Sadigh‐Eteghad, Talebi, Mahmoudi, Babri, & Shanehbandi, 2015; Tanyeri et al., 2015; Wang et al., 2012; Xu et al., 2012).

### 2.4 Statistical analysis

Data were expressed as mean ± *SEM*. All statistical analyses were performed with SPSS (V20.0) software. In the MWM test, mean escape latencies were analyzed using two‐way analysis of variance (ANOVA) with group and time as factors. A standard one‐way ANOVA followed by Tukey HSD test was used for other cases.

## 3 RESULTS

### 3.1 Effects of haloperidol, olanzapine, ziprasidone, and PHA‐543613 on spatial learning and memory in naïve mice in the Morris water maze test

**Figure 1:**
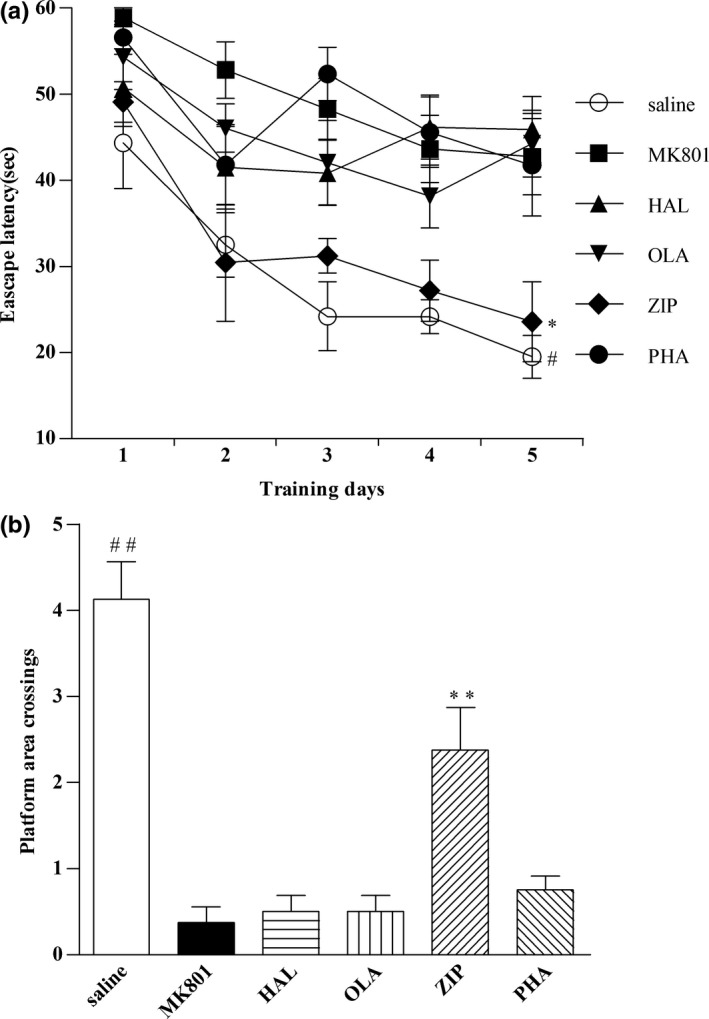
Effects of haloperidol, olanzapine, ziprasidone, and PHA‐543613 on spatial learning and memory in naïve mice in the Morris water maze test. (a) Mean escape latency of reaching the submerged platform in the training period. (b) Crossing platform area times in the probe test. Data were expressed as mean ± *SEM* (*n* = 8/group). *p < .05, **p < .05, compared with MK801, HAL, OLA and PHA group; #*p* < .01, ##*p* < .01, compared with MK801 HAL, OLA and PHA group

Significant differences between groups were detected in mean escape latency and the total number of crossing over the platform area. As shown in Figure 1a, there was a significant difference in mean escape latency between the groups (two‐way ANOVA post‐hoc Turkey's test; *F* (5, 42) = 12.03; *p* < .001). Haloperidol, olanzapine, and PHA‐543613 increased the mean escape latency compared with saline (*p* < .01), whereas ziprasidone decreased the mean escape latency, which showed no significant difference with saline (*p* > .05) indicating that ziprasidone had no influence on water maze performance during the training period in naïve mice.

In probe test, there was a significant difference in the number of platform area crossings between the groups (one‐way ANOVA post‐hoc Dunnett's test; *F* (5, 42) = 23.97; *p* < .001). Ziprasidone increased the total number of platform area crossings compared with haloperidol, olanzapine, and PHA‐543613 (*p* < .05) (Figure 1b).

### 3.2 Effects of haloperidol, olanzapine, ziprasidone, and PHA‐543613 on spatial learning and memory in the Morris water maze test in MK‐801‐treated mice

**Figure 2:**
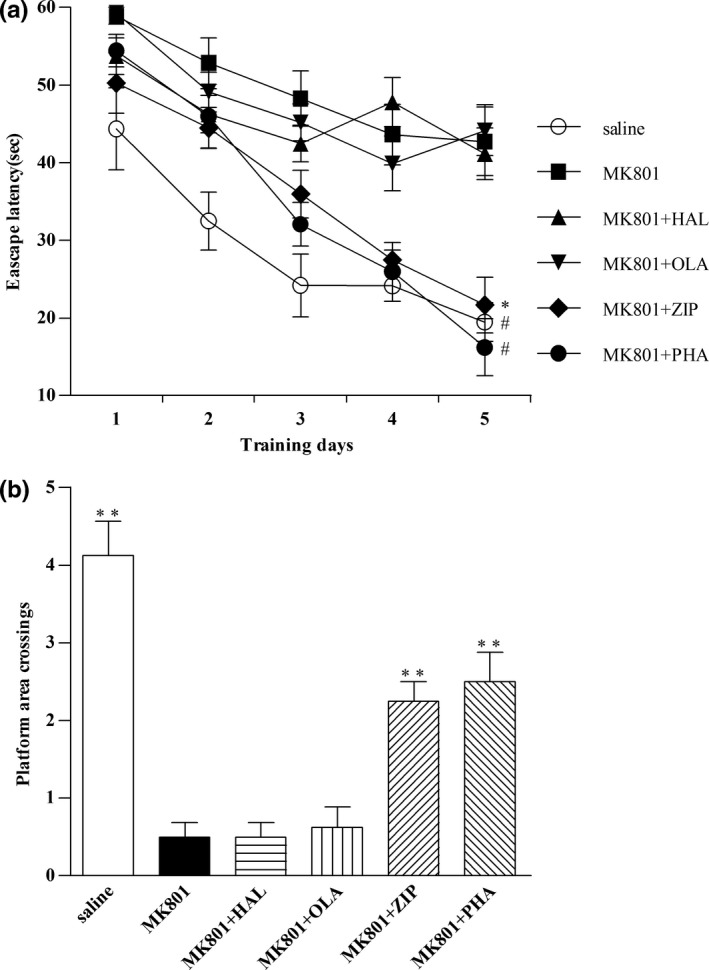
Effects of haloperidol, olanzapine, ziprasidone, and PHA‐543613 on spatial learning and memory in MK‐801‐treated mice in the Morris water maze test. (a) Mean escape latency of reaching the submerged platform in the training period. (b) Crossing platform area times in the probe test. Data were expressed as mean ± *SEM* (*n* = 8/group). *p < 0.05, ^#^ *p* < 0.01, **p < 0.01, compared with MK801, MK801+HAL, and MK801+OLA

Chronic administration of MK‐801 produced spatial learning and memory deficits in mice compared with saline in Morris water maze (*p* < .01). As shown in Figure 2a, haloperidol, and olanzapine increased the mean escape latency compared with saline (*p* < .01), while ziprasidone and PHA‐543613 significantly decreased the mean escape latency compared with haloperidol and olanzapine (*p* < .05; *p* < .01).

In probe test, ziprasidone and PHA‐543613 increased the total number of platform area crossings compared with haloperidol and olanzapine (*p* < .01) (Figure 2b).

### 3.3 Effects of haloperidol, olanzapine, ziprasidone, and PHA‐543613 on swimming speed in naïve and MK‐801‐treated mice

**Figure 3:**
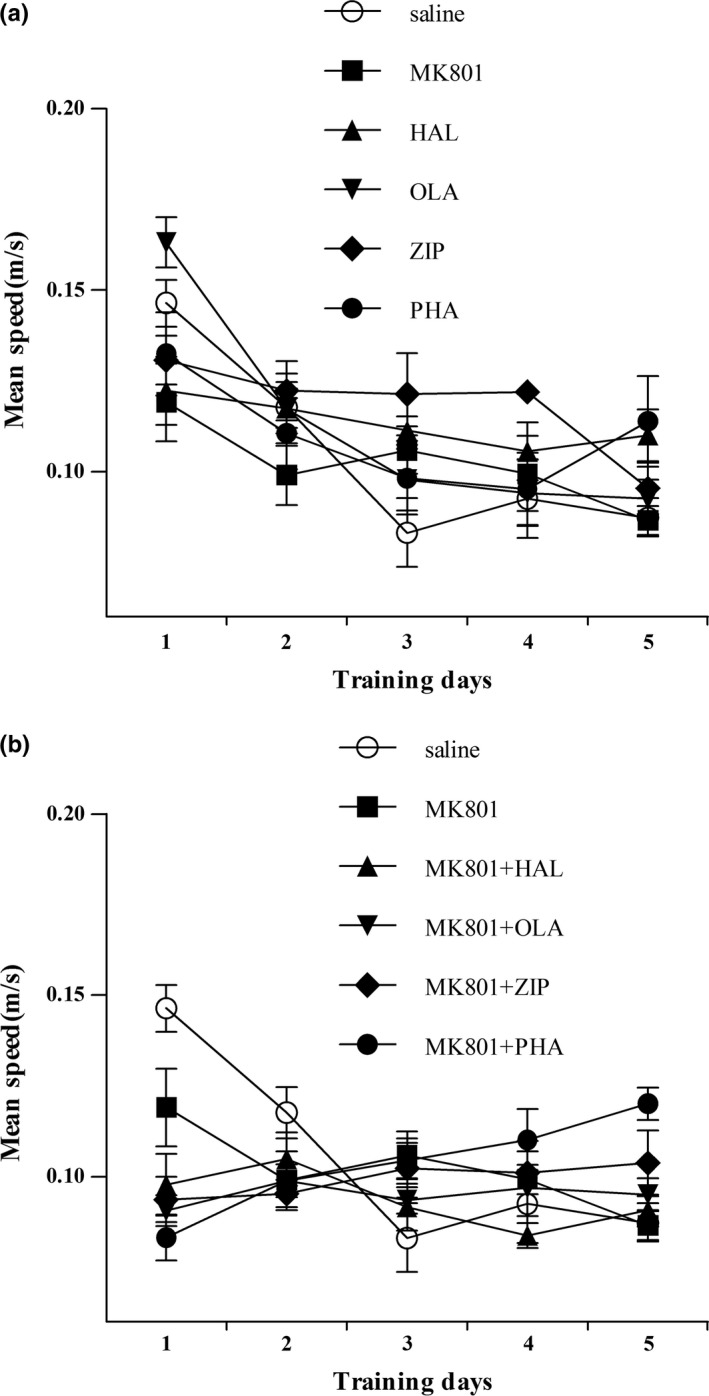
Effects of haloperidol, olanzapine, ziprasidone, and PHA‐543613 on swimming speed in naïve and MK‐801‐treated mice. (a) Swimming speed of naïve mice in training period. (b) Swimming speed of MK‐801‐treated mice in training period. Data were expressed as mean ± *SEM* (*n* = 8/group)

Swimming speed, which is not the necessary factor of cognition, has an effect on mean escape latency (Singh, Kaur, & Sandhir, 2016). As shown in Figure 3a, haloperidol, olanzapine, ziprasidone, and PHA‐543613 had no effect on swimming speed and there was no significant difference between the groups in naïve mice. In MK‐801‐treated mice, haloperidol, olanzapine, ziprasidone, and PHA‐543613 also had no effect on swimming speed (*p* > .05) (Figure 3b).

## 4 DISCUSSION

Cognitive impairment has been recognized as the key component of schizophrenia, and on account of important influence on the outcome of schizophrenia, intensive research has been performed on the evaluation and treatment of cognition impairment (Anderson, McIlwain, Kydd, & Russell, 2015; Brown, Rueter, & Zhang, 2014; Minzenberg & Carter, 2012; Rajagopal, Massey, Huang, Oyamada, & Meltzer, 2014; Yamazaki et al., 2014). Spatial learning and memory as the significant aspect of neurocognition has been a growing recognition in schizophrenia. In previous studies, the effects of the first‐ and second‐generation antipsychotics and alpha‐7 nicotinic receptor acetylcholine agonist on learning and memory were controversial, even the same agent showed different effects in various animal trials (Abdul‐Monim, Reynolds, & Neill, 2003; Hauser et al., 2009; Hoskins, Peeler, Lawson, Barnes, & Ho, 1991; Mutlu et al., 2011).

The MWM test is a widely used approach to investigate spatial learning and memory in mice, which provides a highly reliable form of cognitive evaluation (D'Hooge & De Deyn, 2001; Morris, 1984). During the test, the escape platform was stable. Hence, hippocampal‐dependent spatial reference learning and memory was investigated. In our study, MK‐801 increased the escape latency in acquisition phase as previously mentioned (Mutlu et al., 2011), and decreased the times of crossing the platform area in probe phase. Then, we first observed the influences of agents on spatial learning and memory in naïve mice, and the main finding was that haloperidol, olanzapine, and PHA‐543613 impaired the performance in MWM test, increasing the mean escape latency in acquisition phase and decreasing the number of platform area crossings in probe test. However, ziprasidone seemed to have no impact on mean escape latency and the number of platform area crossings. In one previous study of haloperidol, there was no significant effect on the number of platform area crossings, but the escape latency obviously increased, which led to the thought that the index of platform area crossings was not susceptible to the latency to platform (Xu et al., 2012). Nevertheless, the decrease in the number of platform area crossings that evaluated the accuracy of memory was observed when haloperidol, olanzapine, and PHA‐543613 were treated in naïve mice, which indicated that those agents impaired spatial learning and memory to some degree.

In previous researches, haloperidol and olanzapine disturbed cognitive function in MWM test in naïve mice (Mutlu et al., 2011; Xu et al., 2012), which is consistent with our results. However, PHA‐543613 also performed negative effect on spatial learning and memory in naïve mice. Why PHA‐543613 impaired the MWM performance in naïve mice? On the one side, alpha‐7 nicotinic acetylcholine receptor agonist might produce an anxiogenic effect in mice at the dose which transferred the attention of mice away from escaping on the platform, and the research of the alpha‐7 nicotinic acetylcholine receptor antagonist causing anxiolytic effects backup this point (Irvine, Cheeta, & File, 1999; Roni & Rahman, 2011). On the other side, it is hard to evaluate the effects of the agents that may improve cognitive function in healthy mice.

The hypofunction of NMDA glutamate receptor that can regulate dopamine release in the brain has been considered as a model of schizophrenia (Javitt, 2010). In present study, we create cognitive impairment with subchronic administration of MK801, which has more similarity to the cognitive disorders that manifest in schizophrenia patients. Ziprasidone and PHA‐543613 reversed MK‐801‐induced learning and memory deficits in MWM test, but haloperidol and olanzapine did not. Haloperidol, the typical antipsychotic that blocks dopamine D_2_ receptors, is considered to be poor improvement on cognitive impairment (Gallhofer et al., 2007; Saeedi, Remington, & Christensen, 2006), and it causes obvious extrapyramidal side effects that need anticholinergic which in turn damages cognitive function (Ogino, Miyamoto, Miyake, & Yamaguchi, 2014). It should be noted that haloperidol treatment impaired spatial learning and memory in naïve and had no effect on MK‐801‐induced cognitive deficits, which further showed that blockade of dopamine D_2_ receptors had no benefit on impairment of cognition despite of the significant improvement of positive symptoms.

Olanzapine and ziprasidone belong to the family of atypical antipsychotics that share similar affinity with neurotransmitter receptors, such as 5‐HT_2A_, 5‐HT_2C_, dopamine D_1_, dopamine D_2_ (Arnt & Skarsfeldt, 1998). A previous study (Tyson, Roberts, & Mortimer, 2004) demonstrated that atypical antipsychotics acted on 5HT_2A_ receptors to lead the change of dopamine levels in the prefrontal cortex, which might elucidate the improvement of CIAS. However, in our study, olanzapine disturbed the spatial learning and memory in naïve which is consistent with a previous study (Mutlu et al., 2011). Ziprasidone had no influence on spatial learning and memory in naïve mice, meanwhile it reversed MK‐801‐induced cognitive impairment but olanzapine did not. Although olanzapine and ziprasidone have similar affinity with 5HT_2A_, the 5HT_2A_/D_2_ receptor ration of ziprasidone is higher than olanzapine (Stahl & Shayegan, 2003), which could affect the brain dopamine level and then impact the improvement of cognition. In addition, ziprasidone also acts on 5HT_1A_ receptor as the agonist in human brain tissue, which has been identified as a potential therapeutic target for the treatment of schizophrenia in previous studies (Bantick, Deakin, & Grasby, 2001; Millan, 2000). However, the contribution of 5HT_1A_ receptor in the CIAS is still ambiguous, further study of the mechanism underlying the 5HT_1A_ to improve CIAS is warranted (Meltzer & Sumiyoshi, 2008; Ögren et al., 2008).

Accumulating evidence indicate that alpha‐7 nicotinic acetylcholine receptor, which has a significant effect on cognitive processes, plays an important role in the pathophysiology of schizophrenia and previous studies demonstrated that alpha‐7 nicotinic acetylcholine agonists had emerged as a potential treatment target for the treatment of CIAS (Freedman, 2014; Nikiforuk, Kos, Potasiewicz, & Popik, 2015; Young & Geyer, 2013). PHA‐543613, an alpha‐7 nicotinic partial agonist, has been reported to improve MWM performance in Aβ_25–35_‐induced cognitive deficits mice (Sadigh‐Eteghad et al., 2015). Nevertheless, limited studies investigated the effects of PHA‐543613 on the schizophrenia cognitive deficits. In our study, PHA‐543613 disturbed the performance in naïve mice but improved the spatial cognitive deficits in MK‐801‐treated mice, which implies that the mechanism of alpha‐7 nicotinic acetylcholine agonist affecting cognitive function is complicated.

In our study, we observed the effect of haloperidol, olanzapine, ziprasidone, and PHA‐543613 on the spatial learning and memory. Ziprasidone and alpha‐7 nicotinic agonist PHA‐543613 reversed the MK‐801‐induced cognitive impairment, thus they might be helpful in the treatment of CIAS. However, we did not investigate the cellular and molecular mechanism of the improvement of spatial cognition and change of electrophysiology, which need to be demonstrated in further studies.

## CONFLICT OF INTEREST

None declared.
